# Exacerbation of experimental autoimmune encephalomyelitis in prion protein (PrPc)-null mice: evidence for a critical role of the central nervous system

**DOI:** 10.1186/1742-2094-9-25

**Published:** 2012-01-26

**Authors:** Pauline Gourdain, Clara Ballerini, Arnaud B Nicot, Claude Carnaud

**Affiliations:** 1 INSERM, UMR S 938, Centre de Recherche Hôpital Saint-Antoine, Paris, France; 2 UPMC - Pierre and Marie Curie University (Paris 6), Paris, France; 3 Department of Neurological Sciences, University of Florence, Florence, Italy; 4 INSERM, UMR S 1064, CHU Hôtel-Dieu, 30 Bd Jean Monnet, 44093 Nantes cedex, France; 5 L'UNAM - L'Université Nantes Angers Le Mans, Nantes, France; 6 CHU de Nantes, ITUN, Nantes, France

**Keywords:** Cellular prion protein, Biological function of *Prnp *gene, Gene conservation, PrP knock-out, Experimental autoimmune encephalomyelitis, Bone marrow chimera, Neurodegeneration, Neuroprotection, Glial activation

## Abstract

**Background:**

The cellular prion protein (PrPc) is a host-encoded glycoprotein whose transconformation into PrP scrapie (PrPSc) initiates prion diseases. The role of PrPc in health is still obscure, but many candidate functions have been attributed to the protein, both in the immune and the nervous systems. Recent data show that experimental autoimmune encephalomyelitis (EAE) is worsened in mice lacking PrPc. Disease exacerbation has been attributed to T cells that would differentiate into more aggressive effectors when deprived of PrPc. However, alternative interpretations such as reduced resistance of neurons to autoimmune insult and exacerbated gliosis leading to neuronal deficits were not considered.

**Method:**

To better discriminate the contribution of immune cells versus neural cells, reciprocal bone marrow chimeras with differential expression of PrPc in the lymphoid or in the central nervous system (CNS) were generated. Mice were subsequently challenged with MOG_35-55 _peptide and clinical disease as well as histopathology were compared in both groups. Furthermore, to test directly the T cell hypothesis, we compared the encephalitogenicity of adoptively transferred PrPc-deficient versus PrPc-sufficient, anti-MOG T cells.

**Results:**

First, EAE exacerbation in PrPc-deficient mice was confirmed. Irradiation exacerbated EAE in all the chimeras and controls, but disease was more severe in mice with a PrPc-deleted CNS and a normal immune system than in the reciprocal construction. Moreover, there was no indication that anti-MOG responses were different in PrPc-sufficient and PrPc-deficient mice. Paradoxically, PrPc-deficient anti-MOG 2D2 T cells were less pathogenic than PrPc-expressing 2D2 T cells.

**Conclusions:**

In view of the present data, it can be concluded that the origin of EAE exacerbation in PrPc-ablated mice resides in the absence of the prion protein in the CNS. Furthermore, the absence of PrPc on both neural and immune cells does not synergize for disease worsening. These conclusions highlight the critical role of PrPc in maintaining the integrity of the CNS in situations of stress, especially during a neuroinflammatory insult.

## Background

The prion protein (PrP) is a host-encoded glycoprotein of 30-35 kD molecular weight which presents intriguing physicochemical and biological properties [1]. PrP can adopt at least two conformations: a "cellular" isoform (PrPc) anchored at the membrane by a glycosylphosphatidylinositol (GPI) residue and constitutively expressed on many tissues, and a "scrapie" isoform (PrPSc) rich in beta-pleated sheets, resistant to managed protease treatments and viewed as the propagating agent of human and animal transmissible spongiform encephalopathies (TSE) or prion diseases [2]. Another important feature of PrP is its almost universal expression in vertebrate species including mammalians, birds, amphibians and fish. The molecular structure of PrPc is remarkably conserved in all these species with 2 distinct domains, a random coil flexible one characterized by a series of octarepeats and a globular one containing 3 strictly oriented α-helices [3]. Such evolutionary conservation suggests that the prion protein fulfills ancient and still essential biological functions [4]. Paradoxically, genetically-ablated mice display no major alterations of reproductive, developmental, behavioral, and immunological functions [5,6]. The fundamental role in health of PrPc that justifies its evolutionary conservation is still not elucidated and remains an object of debate [7].

PrPc is most abundantly expressed on neural cells including neurons and glia [8,9], as well as in subsets of cells of hematopoietic origin [10,11]. Therefore, candidate functions have been essentially sought in the immune and central nervous system (CNS). Comparison of PrPc-deficient with PrPc-sufficient mice, or derived cell lines, has provided an extensive, sometimes contradictory and questionable list of candidate functions in relation with cell adhesion, enzymatic activity, signal transduction, copper metabolism, and programmed cell death. In the nervous system, PrPc is candidate for a wide range of functions relevant to protection against ischemic trauma, apoptotic agents and reactive oxygen species [12,13]. PrPc has been also implicated in the preservation of neuronal transmission [14], neurite outgrowth [15], synaptic plasticity [16], circadian rhythm [17] and during aging, maintenance of peripheral myelin [18] as well as motor behavior and memory [19,20].

PrPc fulfills also important functions outside the CNS, in particular in the immune system [21]. The prion protein is promptly upregulated in activated T lymphocytes and is redistributed in lipid rafts, together with signaling molecules [22,23]. The ablation or the masking of PrPc with antibodies reduces T cell proliferation [24,25]. Upon MHC/peptide-driven interactions between T cells and dendritic cells (DC), PrPc migrates at the immunological synapse and exerts differential effects on T cell proliferation and cytokine production, revealed by ablation or antibody masking on the DC or on the lymphocyte side of the synapse [22]. Beside its involvement in adaptive immune responses, PrPc controls various aspects of hematopoietic cell differentiation and function including the self-renewal of bone marrow (BM) progenitors [26], thymic differentiation [27], and the repression of phagocytic activity in macrophages [28]. Beside a direct implication in TSE pathogenesis, as a substrate of transconformation, PrPc has been involved in chronic and acute neurodegenerative conditions including Alzheimer's disease [29] and experimental autoimmune encephalomyelitis (EAE). Three recent and independent studies have reported that clinical and histological manifestations of EAE were worsened in mice which lacked [30,31] or under-expressed PrPc [32]. The authors proposed that PrPc acts as a regulatory molecule and that T lymphocytes that lacked PrPc behaved more aggressively against the CNS than wild type T cells. Alternative explanations such as reduced resistance of neurons to autoimmune insults, or exacerbated aggressiveness of glial cells [33] were either not considered [30,31], or only partially explored [32]. In view of the ongoing debate regarding the biological functions of PrPc, the issue warranted further investigations. To this end, we have engineered reciprocal BM chimeras differentially expressing PrPc in the CNS or in the lymphoid compartment. Our results confirm that EAE is exacerbated in PrPc-deficient mice, but in contrast to the previous studies, they support the conclusion that the origin of disease worsening lies in the absence of PrPc in neural cells and not in lymphoid cells.

## Methods

### Mice

PrPc-ablated (*Prnp*^-/-^) mice were derived from the Zurich I strain [5]. The disrupted gene was backcrossed onto C57BL/6 (B6) mice for more than 12 generations [34]. PrPc-sufficient and PrPc-deficient mice were fully compatible by reciprocal skin grafting (data not shown). They were both kept on CD45.1 and CD45.2 backgrounds. The 2D2 transgene encoding for an α/β T cell receptor (TCR) specific for myelin oligodendrocyte glycoprotein (MOG_35-55_) peptide [35] was backcrossed onto *Prnp*^-/- ^mice for 7 generations. All the mice including CD3ε knock-outs [36] were on a B6 background. Animals were maintained under pathogen-free conditions. All procedures were carried out in strict accordance with the French legislation and European Treaty ETS 123, and were approved by the Ethic Committee of UPMC Univ Paris 6.

### EAE induction and clinical follow up

Adult female mice (8-10 weeks old) were immunized at the base of the tail with 200 μg of MOG_35-55 _peptide (MEVGWYRSPFSRVVHLYRNGK, purity > 85%, NeoMPS, Strasbourg, France) in phosphate buffer saline (PBS) emulsified with an equal part of complete Freund's adjuvant (CFA) supplemented with Mycobacterium tuberculosis H37Ra at 6 mg/ml (Difco Laboratories, Detroit, MI). Mice received in addition 300 ng of pertussis toxin injected intraperitoneally (i.p.) on days 0 and 2 (Calbiochem, Darmstadt, Germany). EAE was also induced in CD3ε knock-out mice adoptively transferred with 3 × 10^6 ^TCR-transgenic CD4+ 2D2 T cells expressing or not PrPc.

EAE-induced mice were scored daily for clinical signs of disease. Scoring was as follows: 0, no detectable signs; 0.5 partial limp tail; 1, complete limp tail; 1.5, limp tail with hindlimb weakness or gait abnormality; 2, limp tail with unilateral hindlimb paralysis; 3, bilateral hindlimb paralysis; 4, bilateral hindlimb paralysis and forelimb weakness; 5, bilateral forelimbs an hindlimbs paralysis (endpoint). Ataxia (disruption of gait and of motor control) was evaluated independently as an index of cerebellar dysfunction. Wild type and *Prnp*^-/- ^controls injected with CFA/PBS plus pertussis toxin remained free of neurological signs.

### Mouse radiation chimeras

Four week-old *Prnp*^+/+ ^and *Prnp*^-/- ^female mice on a CD45.1 background were subjected to 11 Gy total body irradiation, and were intravenously reconstituted 16 hours later with BM cells (20 × 10^6^) prepared from femurs and tibias of CD45.2 gender-matched *Prnp*^+/+ ^and *Prnp*^-/- ^mice. Bactrim (sulfamethoxazole 0.2 mg/mL, trimethoprim 0.04 mg/mL) was added to drinking water for the 15 following days. Ten weeks after reconstitution, mice were immunized with MOG_35-55 _or PBS as described above.

### Flow cytometry analyses

Regulatory T cells were labeled with anti-CD4, anti-CD25, and FoxP3 antibodies as previously described [37]. Lymphoid compartments in reconstituted chimeras were phenotyped with anti-CD4, anti-CD8, anti-CD19 and CD45.1 and CD45.2 antibodies. All antibodies were from BD Bioscience (Le pont de Claix, France). Samples were acquired on a two-laser Becton-Dickinson FACScan and analyzed with BD CellQuest and FlowJo software (TreeStar, Ashland, Oregon, USA).

### Cell-mediated and humoral responses against MOG

Spleen cells from *Prnp*^+/+ ^and *Prnp*^-/- ^mice were collected at 22 days post-immunization (dpi). CD4+ T cells enriched by negative magnetic selection (Dynabeads, Invitrogen, France) were assayed for antigen-specific proliferation and lymphokine release. Spleen DC serving as a source of antigen-presenting cells (APC) were isolated from non-immunized wild type mice by positive selection on a CD11c magnetic kit (Miltenyi Biotec, Paris, France). CD4+ T cells (2 × 10^5^) were plated together with 3 × 10^4 ^DC per well. MOG peptide was added at 100, 10, 1 and 0 μg/ml. Proliferation was measured by thymidine incorporation after 4-day culture, as previously described [34]. Lymphokine release was assessed in supernatants from replicate cultures collected after 48 h. Concentrations of IFN-γ and IL-17 in culture supernatants were measured by solid phase ELISA kit according to the manufacturer recommendations (PeproTech, Neuilly, France).

Anti-MOG antibodies were titrated by ELISA in sera from MOG-induced mice at 22 dpi according to standard procedures [34]. Serum dilutions ranging from 1:50 to 1:2500 were distributed into polystyrene 96 well ELISA plates (Maxisorp, Nunc, Denmark) pre-coated with MOG_35-55 _peptide. Bound antibody was revealed with goat anti-mouse IgM + IgG + IgA (H + L) immunoglobulin conjugated to alkaline phosphatase (Southern Biotech, USA) and p-nitrophenyl phosphate as substrate. Absorbance was measured at 405 nm. Results are reported as optical density (OD).

### Histology

A series of 6-8 five micrometer thick coronal sections of paraffin embedded spinal cord (lumbar region) or cerebellum samples were stained with hematoxylin and Bielschowsky silver impregnation to evaluate inflammatory infiltrates and axonal loss respectively [38]. The number of perivascular inflammatory foci per section was first scored on spinal cords from *Prnp*^+/+ ^and *Prnp*^-/- ^mice by two researchers blinded to the genotype. Because differences in this inflammatory score were not evidenced, the total number of white matter nuclei/spinal cord section was also evaluated to assess the extent of white matter immune cell infiltration. Histological scores of axonal loss for individual animals were evaluated blindly using a semi-quantitative grid where 0 is no disease and ascending numerical score indicate increasing degrees of pathology.

### Immunohistochemistry

Dewaxed sections were subjected to heat antigen retrieval in 10 mM Na-Citrate buffer (pH 6.0). After preincubation in 30% donkey normal serum and 0.05% Triton-X in PBS, sections were incubated overnight at 4°C with primary antibody at optimized working dilution. Mouse monoclonal antibody to plasma membrane calcium ATPase 2 (PMCA2) (1:500, BD Biosciences, San Jose, CA) used to visualize Purkinje neurons and their processes was revealed with a biotinylated secondary anti-mouse IgG (F(ab')2 Ab, 1:200; Jackson ImmunoResearch) for 1 h, and a streptavidin-peroxidase complex (Perkin-Elmer Life Sciences, Boston, MA) at 1:200 for 1 h plus tyramide-fluorescein (1:100 for 10 min; PerkinElmer Life Sciences). Polyclonal rabbit antibody to glial fibrillary acidic protein (GFAP) (DakoCytomation, Glostrup, Denmark) was used at 1:5000 to detect astrocytes. Microglia/macrophages were visualized by ionized calcium-binding adapter molecule-1 (Iba-1) rabbit antibody (1:500; Wako Chemicals, Richmond, VA, Wako, Japan). The DyLight568-coupled F(ab')2 secondary (anti-rabbit IgG, 1:200; Jackson ImmunoResearch, Suffolk, England) was used. Sections were coverslipped using Vectashield mounting medium with DAPI (Vector Laboratories, Burlingame, CA). The analysis of negative controls (omission of primary antibody) was simultaneously performed in order to exclude the presence of non-specific immunofluorescent staining, cross-immunostaining, or fluorescence bleed-through. A Nikon Eclipse E660 microscope with appropriate excitation and emission filters for each fluorophore was used to acquire representative images from the examined specimens. Images were acquired with × 20 to × 40 objectives using a digital camera (DMX1200, Nikon) driven by NikonACT-1 software and were processed for printing using Adobe Photoshop.

### Statistical analysis

Comparisons between two groups were performed with non-parametric Mann-Whitney test; comparisons between 3 groups were submitted to non-parametric Kruskal-Wallis variance analysis followed by two-by-two Dunn's comparison tests. Differences were considered significant at *p *< 0.05. Analyses were performed with GraphPad software (San Diego, CA, USA).

## Results

### PrPc ablation increases EAE clinical severity, duration and associated neurohistopathological parameters

**Figure 1:**
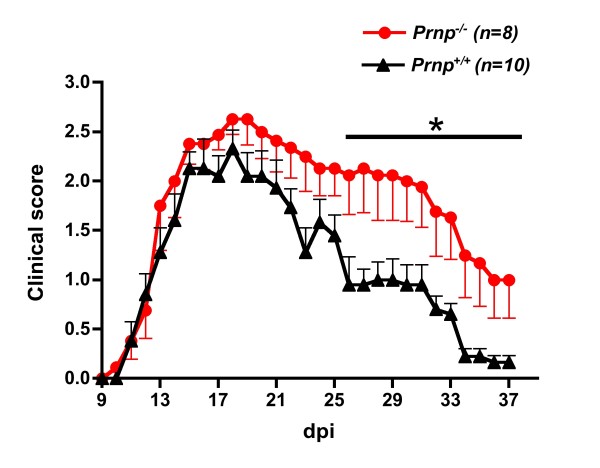
**Exacerbated EAE in PrPc-ablated (*Prnp*^-/-^) mice**. Clinical scores (mean ± SEM) in PrPc-deficient (black dots) versus PrP-sufficient (black triangles) mice. Disease onset was identical in the two groups (*Prnp*^-/-^: 12.7 ± 0.3; *Prnp*^+/+^: 12.4 ± 0.4). Clinical scores began significantly diverging at 22 dpi after the initial peak of disease. *: *p *< 0.05 by Mann-Whitney (n = 8-10/group).

**Figure 2:**
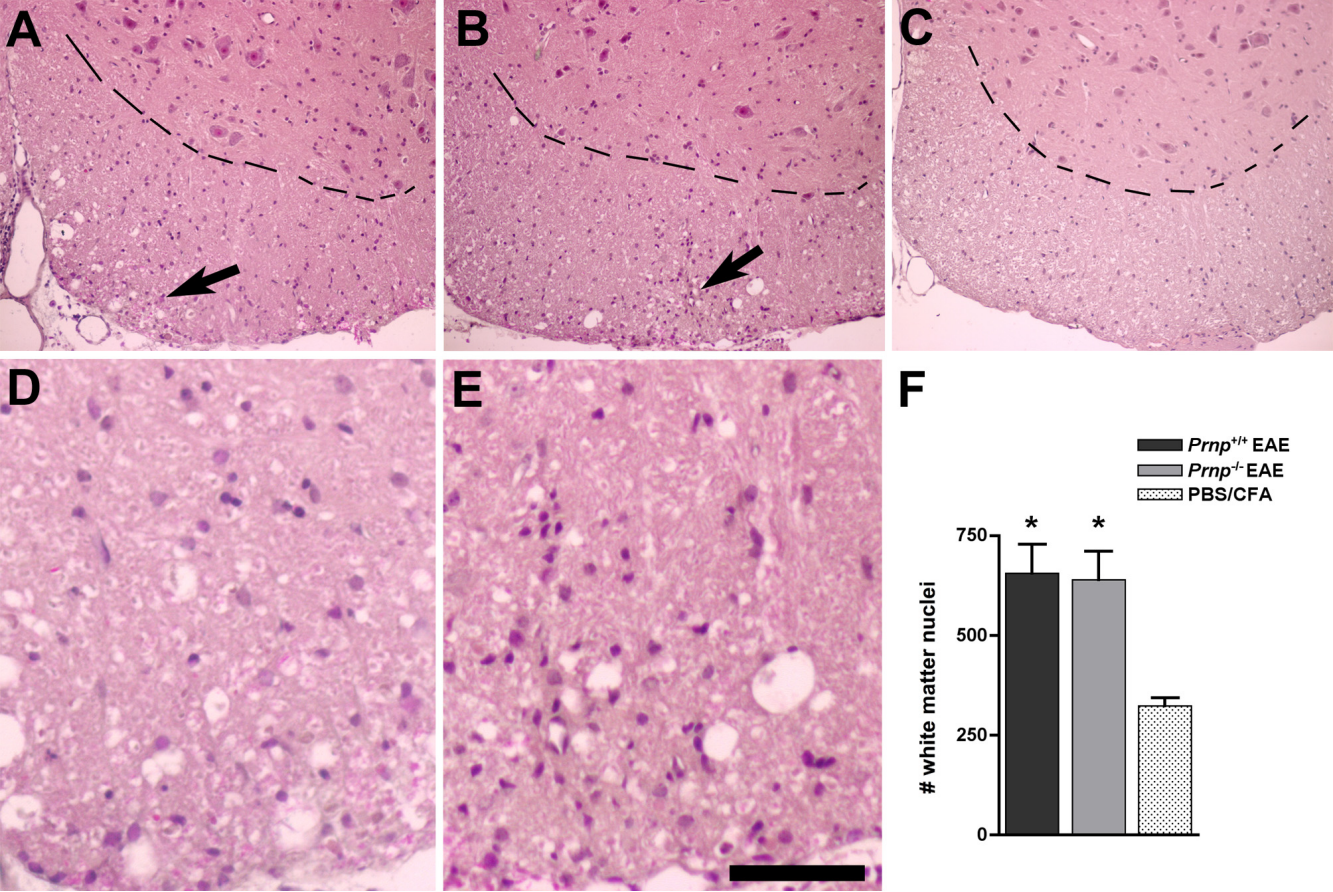
**No difference in mononucleated cell infiltration between EAE-induced *Prnp*^-/- ^and *Prnp*^+/+ ^mice**. Hematoxylin stained sections of ventral spinal cord at 22 dpi; (**A**) *Prnp*^+/+ ^EAE-induced mouse; (**B**) *Prnp*^-/- ^EAE-induced mouse and (**C**) non-EAE PBS/CFA control. (**D**) and (**E**) white matter sections of a *Prnp*^-/- ^PBS control and a *Prnp*^-/- ^EAE mouse respectively. Scale bar: A-C, 200 μm; D-E, 56 μm. (**F**) average numbers of white matter nuclei per spinal cord section of *Prnp*^+/+ ^and *Prnp*^-/- ^EAE mice and of PBS controls (n = 3/group). *: *p *< 0.001 versus controls (Dunn's post test analysis).

**Figure 3:**
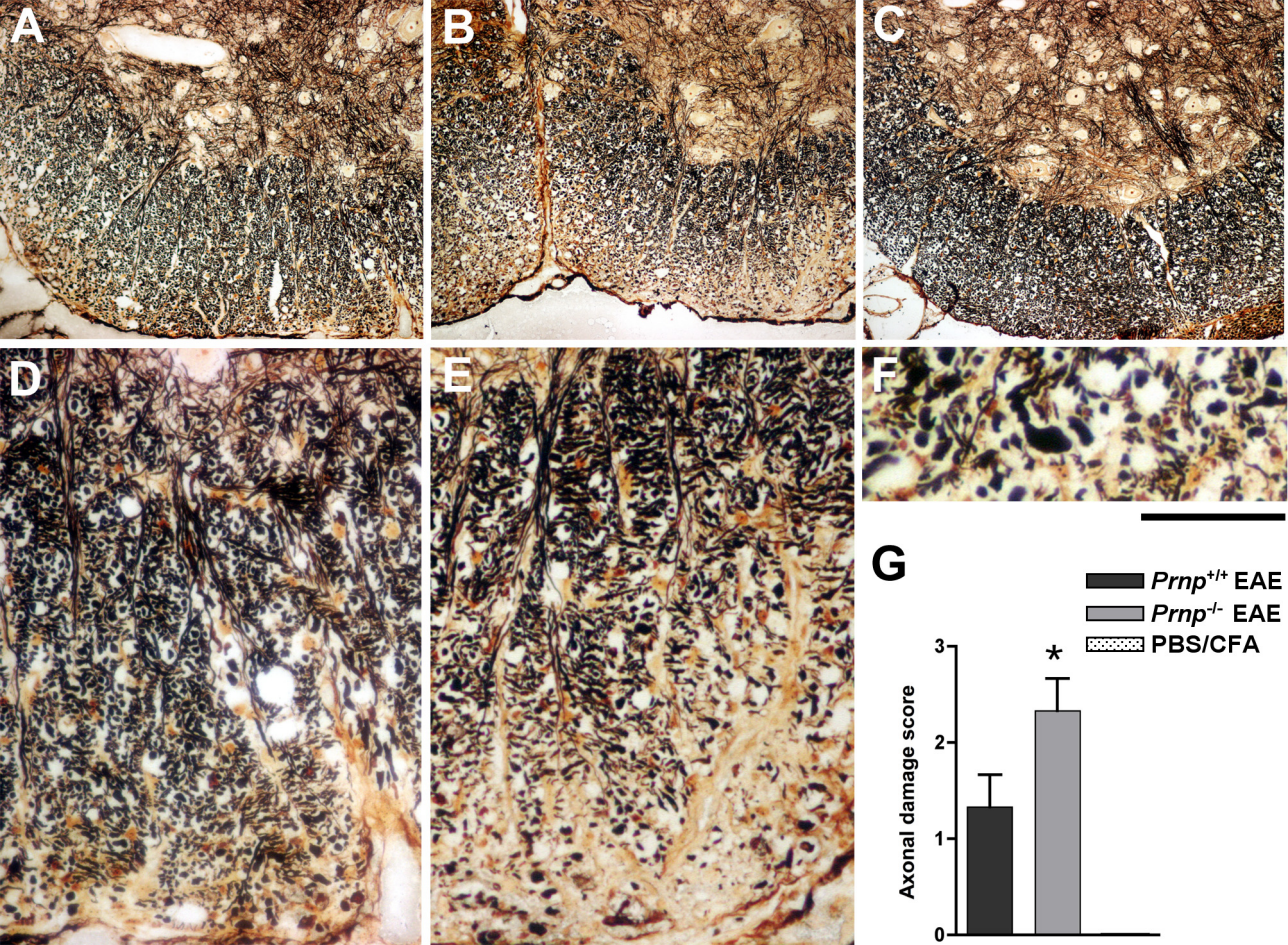
**More severe axonal loss/damage in the white matter of *Prnp*^-/- ^mice**. Sections of ventral spinal cord **r**evealed by Bielschowsky's silver staining; (**A**) *Prnp*^+/+ ^EAE-induced mouse; (**B**) *Prnp*^-/- ^EAE-induced mouse and (**C**) non-EAE PBS/CFA control. (**D**) and (**E**) focus into the white matter of ventral spinal cord from a *Prnp*^+/+ ^EAE mouse and a *Prnp*^-/- ^EAE mouse, respectively. (**F**) higher magnification in the damaged white matter of a *Prnp*^-/- ^EAE mouse showing typical axonal bulb formation. Scale bars: A-C: 200 μm, D-E: 30 μm, F: 15 μm. (**G**) quantitative evaluation of axonal damage in *Prnp*^+/+ ^versus *Prnp*^-/- ^EAE-induced mice, and wild type PBS controls (n = 3/group). *: *p *< 0.05 vs. PBS controls (Dunn's post test analysis).

The increased severity of EAE clinical symptoms in mice lacking PrPc was confirmed in 3 consecutive experiments involving a total of 15 *Prnp*^-/- ^and 16 *Prnp*^+/+ ^mice. As shown in Figure 1 representative of one of these experiments, after a similar initial phase extending up to 15 dpi, clinical scores increased faster in the *Prnp*^-/- ^group and remained higher. Differences reached statistical significance at 24 dpi and remained significant till 37 dpi (*p *< 0.05 by Mann-Whitney test). EAE was not only more severe, but it resolved also more slowly as reported in other studies. Histological analyses of spinal cords were done in a subset of mice (n = 3/group) sacrificed at 22 dpi, the earliest time point where clinical scores started diverging significantly when analyzing all mice. As shown in Figure 2, infiltration of the spinal cord by immune cells was similar in *Prnp*^+/+ ^and *Prnp*^-/- ^mice in spite of the more severe neurological symptoms in the latter group. Quantitative microscopic examinations of hematoxylin stained sections showed twice as many nuclei in the white matter of spinal cord sections in MOG-challenged mice than in PBS controls (*p *< 0.001 by variance analysis). But there was no significant difference between MOG-challenged *Prnp^-/- ^*and *Prnp^+/+ ^*mice (Figure 2F). In contrast, Bielschowky's staining revealed increased axonal damage during EAE in *Prnp*^-/- ^mice compared to *Prnp*^+/+ ^mice (Figure 3).

Most strikingly, examination at high magnification showed the presence of enlarged axonal bulbs in *Prnp*^-/- ^mice, a sign of severe damage (Figure 3F). Differences between *Prnp^-/- ^*MOG-challenged mice and PBS controls were significant by Dunn's comparison test (*p *< 0.05), though differences between *Prnp^-/- ^*and *Prnp^+/+ ^*EAE mice did not reach *p *value < 0.05 (Figure 3G).

### Increased EAE severity in PrPc-deprived mice is not associated with abnormal anti-MOG immune responses

**Figure 4:**
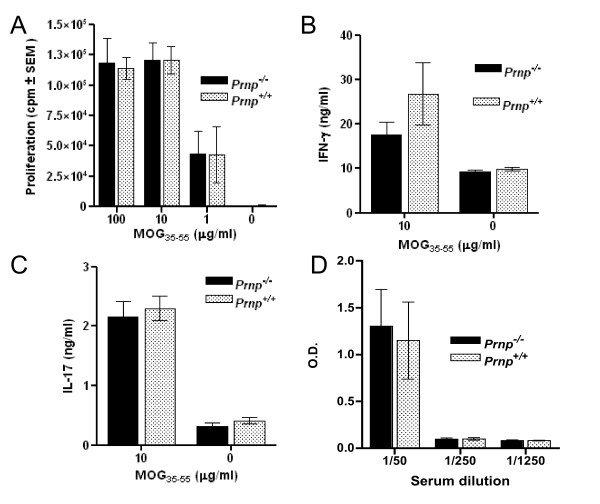
**CD4+ T cells from *Prnp*^-/- ^and *Prnp*^+/+ ^EAE-induced mice respond similarly to MOG_35-55_**. (**A**) In vitro proliferation of purified CD4+ T cells from 3 *Prnp*^-/- ^and 3 *Prnp*^+/+ ^primed mice tested individually. Thymidine incorporation was measured after 92 hours of culture. (**B**) and (**C**) concentrations of IFN-γ and IL-17 in supernatants collected at 48 hour of culture of MOG-primed CD4+ T cells in vitro reactivated with APC and MOG peptide. (**D**) anti-PrP antibodies titrated by solid phase ELISA in individual sera of EAE-induced mice collected at 22 dpi.

Given the implication of PrPc in immune responses, notably through T-DC interactions [22], we tested the possibility that the lack of PrPc on T cells may modify their responses against myelin antigens, resulting in more dramatic EAE manifestations as suggested in the reported studies [30-32]. T cells from MOG-primed *Prnp*^-/- ^and *Prnp*^+/+ ^mice were thus collected between 18 and 24 dpi, at the peak of EAE exacerbation, and assayed for antigen-specific proliferation and cytokine release. Serum samples collected at the same time were used for antibody titration. As indicated in Figure 4A, T cells proliferated to the same extent at all the concentrations of MOG tested, irrespective of whether they were PrPc-deficient or PrPc-sufficient, and despite marked differences in clinical scores of those mice. In addition, the frequencies of T cells secreting IFN-γ and IL-17, two cytokines of major importance in EAE pathogenesis, were not significantly different (Figure 4B-C). Significant amounts of circulating antibody against MOG were detected at 1/50 serum dilution in EAE-induced mice, with no difference between wild type and PrPc-ablated mice (Figure 4D). Sera of normal non-EAE-induced mice were totally devoid of anti-MOG antibody (data not shown). In addition, we verified that the number of regulatory CD25+/FoxP3+ T cells which express high levels of PrPc under normal conditions [39], was not reduced in PrPc-deficient mice (data not shown). In conclusion, we could not find any indication that immune responses against the myelin-derived self antigen were modified, 22 days after immunization, as a consequence of PrPc ablation.

### PrPc-deficient anti-MOG CD4+ T cells are not more encephalitogenic than PrPc-sufficient controls

Although we did not find evidence to support the idea that CD4+ T cells lacking PrPc adopted a stronger inflammatory profile and were therefore more deleterious than wild type T cells, we verified directly this hypothesis by comparing disease development in CD3ε^-/- ^mice adoptively transferred with anti-MOG T cells expressing or not PrPc.

**Figure 5:**
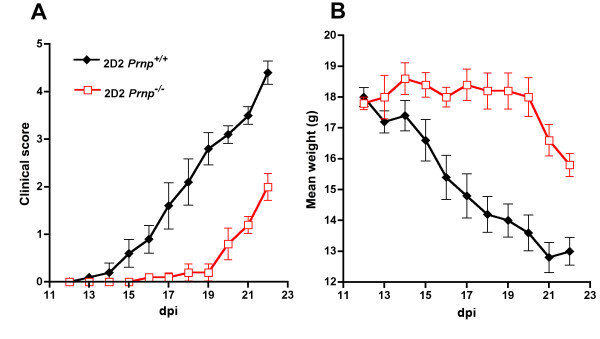
**Less severe EAE in CD3ε^-/- ^mice adoptively transferred with PrPc-deficient 2D2 T cells**. (**A**) clinical scores (mean ± SEM). All the mice transferred with PrPc-expressing effector T cells reached terminal stage disease (mean score 4.5) at 20 dpi whereas the average score in the recipients of PrPc-deficient T cells was 1.7 at the same time point. (**B**) Corresponding weight loss in recipients of PrPc-expressing or deficient 2D2 T cells (mean ± SEM).

CD4+ T cells from the anti-MOG_35-55 _2D2 TCR transgenic line [35] respectively backcrossed to *Prnp*^-/- ^or to *Prnp*^+/+ ^B6 mice, were transferred into CD3ε^-/- ^recipients challenged with MOG_35-55 _peptide plus pertussis toxin. As shown in Figure 5A-B and in Table 1, 2D2 T cells lacking PrPc far from being more encephalitogenic caused a milder disease with a delayed onset and lower clinical scores and with less weight loss and no ataxia compared to the PrPc-sufficient counterparts.

**Table 1:** Ataxia incidence during EAE in mice adoptively transferred with encephalitogenic TCR-transgenic 2D2 T cells and in BM chimeras

Mice	Ataxia
[2D2 *Prnp*^+/+ ^> CD3ε^-/-^]	2/5

[2D2 *Prnp*^-/- ^> CD3ε^-/-^]	0/5

[*Prnp*^+/+ ^*> Prnp*^-/-^]	5/7

[*Prnp*^-/- ^*> Prnp*^+/+^]	0/7

### Exacerbation of EAE is due to the absence of PrPc in neural cells rather than in immune cells

To determine the neural versus immune origin of EAE exacerbation in mice lacking PrPc, we generated reciprocal [*Prnp^-/- ^*>*Prnp^+/+^*] and [*Prnp*^+/+ ^>*Prnp*^-/-^] BM chimeras. This strategy has been widely used in EAE studies to establish the tissue origin of effector molecules that are ubiquitously expressed in the CNS and the lymphoid system [40-42]. To control for an effect of total body irradiation, a group of mice was lethally irradiated and restored with homologous *Prnp*^-/- ^or *Prnp*^+/+ ^BM. PBMC were phenotyped at 10 weeks post-transfer. T and B cell subsets were normally reconstituted and more than 90% of CD4+ T cells were of donor origin (Table 2). Residual chimerism was the same irrespective of donor/recipient combinations.

**Table 2:** Hematopoietic reconstitution of irradiated CD45.1^+ ^mice by CD45.2^+ ^BM

[*Prnp^-/- ^> Prnp^+/+^*]	CD19+		CD4+		CD8+	
	
	CD45.1+	CD45.2+	CD45.1+	CD45.2+	CD45.1+	CD45.2+
#1	0.1	65.04	2.34	16.95	1.64	8.53

#2	0.14	57.56	2.86	18.34	2.28	11.45

#3	0.07	60.94	1.83	19.04	2.45	10.21

#4	0.04	64.02	2.31	17.24	1.79	8.86

#5	0.08	59.17	3.33	19.93	1.8	9.82

#6	0.03	65.27	2.86	17.31	1.57	8.41

#7	0.09	58.02	4.23	19.54	2.84	10.18

**[*Prnp*^+/+ ^*> Prnp*^-/-^]**	**CD19+**		**CD4+**		**CD8+**	
	
	CD45.1+	CD45.2+	CD45.1+	CD45.2+	CD45.1+	CD45.2+

#1	0.05	52.13	3.41	20.11	2.98	10.61

#2	0.09	56.37	3.59	21.66	3.1	10.46

#3	0.12	60.21	3.97	19.15	3.26	9.62

#4	0.15	59.91	3.89	18.88	3.28	9.83

#5	0.11	56.15	4.24	20.15	3.32	9.68

#6	0.2	60.52	3.27	17.23	2.59	8.88

#7	0.15	47.91	3.58	18.44	3.18	10.02

**[*Prnp*^+/+ ^*> Prnp*^+/+^]**	**CD19+**		**CD4+**		**CD8+**	
	
	CD45.1+	CD45.2+	CD45.1+	CD45.2+	CD45.1+	CD45.2+

#1	0.08	65.16	2.24	18.17	1.5	9.07

#2	0.08	67.21	1.92	17.3	1.35	8.29

#3	0.04	63.49	1.3	16.96	0.7	8.88

#4	0.02	69.12	2.52	15.95	1.29	7.25

**[*Prnp*^-/- ^*> Prnp*^-/-^]**	**CD19+**		**CD4+**		**CD8+**	
	
	CD45.1+	CD45.2+	CD45.1+	CD45.2+	CD45.1+	CD45.2+

#1	0.12	52.03	5.18	22.91	3.46	11.66

#2	0.09	54.22	3.93	19,76	3.54	12.3

#3	0.12	57.48	4.3	20.02	2.82	11.06

PBMC samples were phenotyped 10 weeks post-transfer by cell-surface staining. Lymphocyte percentages were assessed by flow cytometry

**Figure 6:**
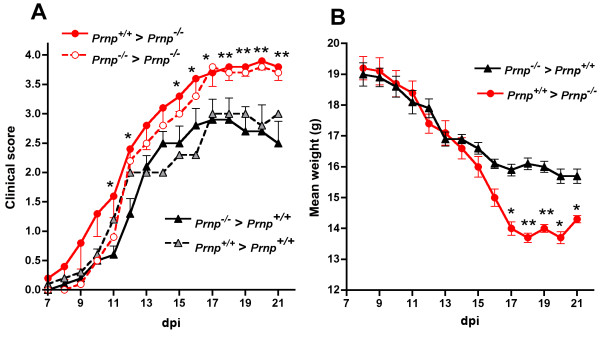
**More severe EAE in chimeric mice with PrPc-depleted CNS cells**. (**A**) clinical scores in reciprocal BM chimeras (continuous lines, mean ± SEM) and irradiation controls restored with homologous BM (dashed lines, mean). Clinical scores begin diverging at 11 dpi. *: *p *< 0.05; **: *p *< 0.01 by Mann-Whitney. (**B**) Corresponding weight loss (mean ± SEM) associated to EAE exacerbation in [*Prnp*^+/+ ^>*Prnp*^-/-^] chimeras. Mann-Whitney statistical difference between [*Prnp*^+/+ ^>*Prnp*^-/-^] vs. [*Prnp*^-/- ^>*Prnp*^+/+^] chimeras *: *p *< 0.05; **: *p *< 0.01.

Total body irradiation exacerbated manifestation of EAE in all the mice as a probable consequence of increased blood brain barrier permeability and glial activation [43]. Neurological deficits started between 8 and 10 dpi in all the groups, but differences between chimeras were observed (Figure 6A). EAE was more severe in [*Prnp*^+/+ ^>*Prnp*^-/-^] than in [*Prnp*^-/- ^>*Prnp*^+/+^] mice (*p *< 0.05 and 0.01 by Mann-Whitney test). Loss of body weight (Figure 6B) and ataxia (Table 1), two parameters of disease severity and of cerebellum dysfunction respectively, were also more pronounced in [*Prnp*^+/+ ^>*Prnp*^-/-^] mice than in [*Prnp*^-/- ^>*Prnp*^+/+^] mice (*p *< 0.05 and 0.01 by Mann-Whitney test). Irradiation controls, reconstituted with homologous BM, reproduced the same pattern of disease severity as heterologous chimeras (Figure 6A) confirming that differences between chimeras were not an artifact resulting from heterologous BM grafting. Interestingly, disease severity was comparable in [*Prnp^-/- ^*>*Prnp^-/-^*] and in [*Prnp^+/+ ^*>*Prnp^-/-^*] mice suggesting that the absence of PrPc in the hematopoietic compartment did not aggravate further the symptoms induced by the absence of PrPc in the CNS.

### Cerebellar alterations associated with EAE exacerbation in [*Prnp*^+/+ ^>*Prnp*^-/-^] chimeras

**Figure 7:**
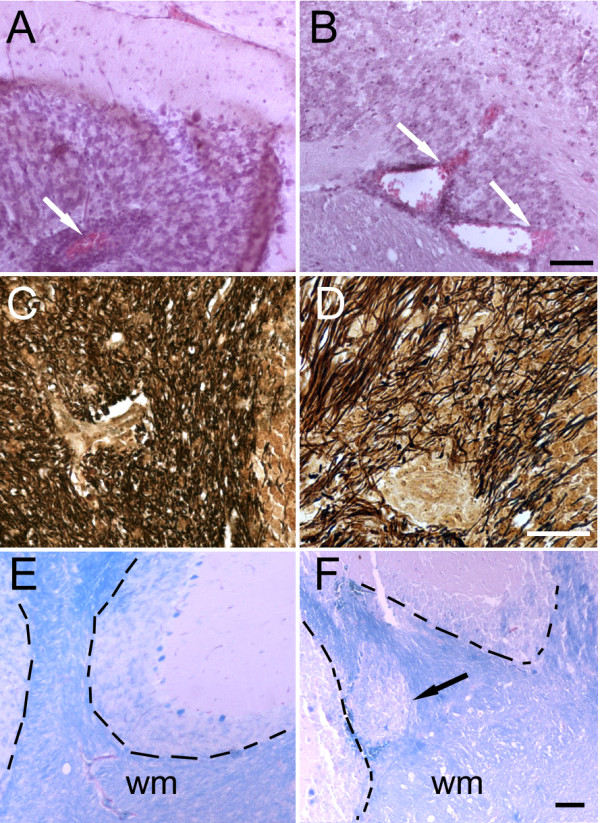
**Representative histological stainings from cerebella of chimeric mice with EAE**. Left panels, [*Prnp*^-/- ^>*Prnp*^+/+^] mouse. Right panels, [*Prnp*^+/+ ^>*Prnp*^-/-^]. (**A-B**) hematoxylin/eosin staining showing white matter perivascular cellular infiltrates (black arrows) that were more spread in [*Prnp*^+/+ ^>*Prnp*^-/-^] than in [*Prnp*^-/- ^>*Prnp*^+/+^] mice. (**C-D**) Bielschowsky silver impregnation staining in the white matter, showing reduced axonal staining in [*Prnp*^+/+ ^>*Prnp*^-/-^] mouse as compared to [*Prnp*^-/- ^>*Prnp*^+/+^] mouse, despite the presence of an inflammed vessel in the center in both examples. (**E-F**) Luxol Fast blue staining showing a clear-cut demyelinating area (white arrow) in [*Prnp*^+/+ ^>*Prnp*^-/-^] mouse as compared to the normal appearing white matter in [*Prnp*^-/- ^>*Prnp*^+/+^] mouse. The dashed lines point to the white matter/grey matter limit. wm, white matter. Scale bars: 100 μm.

**Figure 8:**
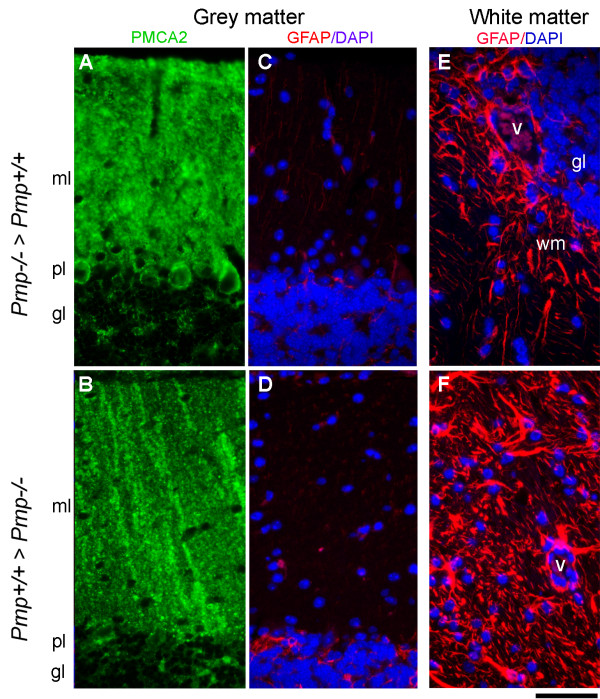
**Representative immunostaininings for PMCA2 and GFAP in the cerebellum of chimeric mice with EAE**. Double indirect immunofluorescence for PMCA2 and GFAP followed by DAPI staining was performed on cerebellar sections from [Prnp^-/- ^> Prnp^+/+^] or [Prnp^+/+ ^> Prnp^-/-^] mice with EAE. (**A-B**) PMCA2 immunofluorescence and (**C-D**) GFAP/DAPI stainings in corresponding section of the grey matter. (**E-F**) GFAP/DAPI stainings in the white matter (cerebellar peduncle). ml, molecular layer of the grey matter; pl, Purkinje layer of the grey matter; gl, granular layer of the grey matter; v, blood vessel; wm, white matter. Scale bar, 100 μm.

**Figure 9:**
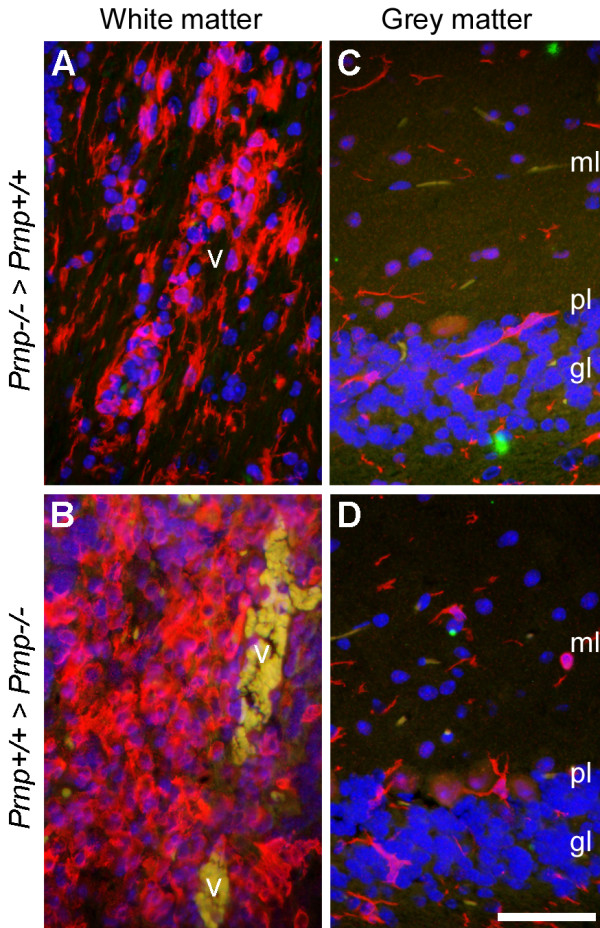
**Representative immunostainings for Iba-1 in the cerebellum of chimeric mice with EAE**. Immunofluorescence for Iba-1 (red) followed by DAPI staining was performed on cerebellar sections from [Prnp^-/- ^> Prnp^+/+^] or [Prnp^+/+ ^> Prnp^-/-^] mice with EAE. (**A-B**) white matter (cerebellar peduncle). (**C-D**) cerebellar cortical grey matter. Green/yellow labeling is due to autofluorescence by remaining erythrocytes in some blood vessels. ml, molecular layer of the grey matter; pl, Purkinje layer of the grey matter; gl, granular layer of the grey matter; v, blood vessel. Scale bar, 100 μm.

The manifestation of severe ascending forms of EAE leading in many cases to ataxia warranted histological evaluation of the cerebellum. EAE predominantly targets the spinal cord but can affect the cerebellar peduncles [44]. Tissue samples from [*Prnp*^+/+ ^>*Prnp*^-/-^] and [*Prnp*^-/- ^>*Prnp*^+/+^] mice were collected at the peak of the active disease phase in order to assess immune cell infiltration, myelin degeneration, axonal damage and glial activation. EAE exacerbation in [*Prnp^+/+ ^*>*Prnp^-/-^*] was not correlated with more massive infiltration of the white matter by immune cells. Perivascular cellular infiltrates were abundant in both combinations of chimeras. However, foci appeared more extended in [*Prnp^+/+ ^*>*Prnp^-/-^*] mice which developed an exacerbated disease (Figure 7A-B). As anticipated, cerebella from [*Prnp*^+/+ ^>*Prnp*^-/-^] chimeras displayed more severe axonal damage revealed by Bielschowsky's axonal staining of white matter tracts (Figure 7C-D). Areas of demyelination revealed by Luxol fast blue staining were also more extended in these mice (Figure 7E-F). As an additional parameter of neuronal damage, we looked at the downregulation of plasma membrane calcium ATPase 2 (PMCA2) whose function is to extrude calcium from neurons and whose expression is impaired in EAE and spinal cord trauma [45,46]. In agreement with the above observations, loss of PMCA2 immunoreactivity in Purkinje cell bodies and their dendritic arborization in the molecular layer was more pronounced in [*Prnp*^+/+ ^>*Prnp*^-/-^] than in [*Prnp*^-/- ^>*Prnp*^+/+^] mice indicating that Purkinje neuronal damage correlates with disease severity in radiation chimeras (Figure 8A-B). Astrocytosis and microgliosis were evaluated by GFAP and Iba-1 staining respectively. Astrogliosis was observed in the cerebellar white matter of all chimeras, and occurred especially around inflamed vessels, where numerous hypertrophic GFAP positive cells were present. Still, astrocytes in [*Prnp*^+/+ ^>*Prnp*^-/-^] chimeras presented more intense immunoreactivity and thicker processes, both in the grey matter (Figure 8C-D), and in the white matter (Figure 8E-F). Immunoreactivity for Iba-1, a marker of blood-borne macrophages and resident microglia, was also observed in the cerebellar white and grey matter of both types of chimeras (Figure 9). However, as for astrogliosis, Iba-1 immunoreactivity on macrophage-like cells was clearly much higher in the white matter of [*Prnp*^+/+ ^>*Prnp*^-/-^] especially around inflamed vessels (Figure 9B) indicating increased neuroinflammation in cerebellar parenchyma. In the grey matter, Iba-1 staining was restricted to resting and scattered activated microglial cells (with thick processes), especially in the granule cell layer of both types of chimeras (Figure 9C-D). In conclusion, clinical exacerbation observed in chimeras in which the CNS was PrPc-depleted correlated with more severe parenchymal damage and increased glial activation in cerebellum.

## Discussion

### Lack of evidence supporting T cell implication in EAE exacerbation

The common denominator to the multiple functions attributed to PrPc is still elusive. In the immune system, PrPc is viewed as a molecule of activation and regulation of adaptive immune responses, whereas in the CNS, PrPc maintains the physiological integrity of the system and protects neurons against various insults [7]. It is therefore crucial to dissect the specific contribution of the prion protein in manifestations such as EAE which involve the neural and immune systems. As previously reported [30-32], and as confirmed in the present study, mice deprived of PrPc by genetic ablation or by RNA interference develop worsened clinical and histopathological manifestations of EAE. At variance with Tsutsui et al. and Hu et al., we did not observe an acceleration of disease onset or increased leukocyte infiltration of the white matter. But in agreement with the other studies [30-32], neurological scores were significantly higher in PrPc-deprived mice and remissions were clearly delayed. Accordingly, axonal damage, revealed by Bielschowky's staining was more pronounced in the spinal cords of PrPc-deprived mice.

In view of our own results suggesting that PrPc exerts a control on T cell responses [22], and of those reported by Tsutsui et al. [31], Ingram et al. [30] and Hu et al. [32], we anticipated to find differences in the responses to MOG peptide of PrPc-deficient T cells versus wild type T cells. Surprisingly, T cells derived from *Prnp*^-/- ^mice and challenged with MOG_35-55 _peptide proliferated to the same extent as controls, produced the same amounts of IFNγ and IL-17 cytokines, two major cytokines involved in EAE pathogenesis, provided similar help to B cells for antibody production, and contained identical proportions of regulatory CD25+/FoxP3+ T lymphocytes which highly express PrPc [39]. The present discrepancies may be the result of the heterogeneity in mouse strains, of the variety of encephalitogenic antigens, and of the protocols for T cell assays. Even within the reported studies, observations diverged with respect to the robustness of cytokine responses in *Prnp*^-/- ^mice and to the hierarchy between Th1 and Th17 inflammatory T cells [30]. Our results being based on a single PrP knock-out line backcrossed on a B6 genetic background, we cannot exclude the possibility that the discrepancies observed with the above mentioned studies result from the use of different PrPc-ablated strains on different genetic backgrounds [30,31], or from differences in the methodology of gene silencing [32]. An interesting possibility of divergence with respect to the latter study could be that at variance with pharmacological ablation by small interfering ribonucleic acid (siRNA) which is limited in time and in extension, genetic knockdown generates long-term immunological compensatory mechanisms. This eventuality cannot be formally excluded, even though no such compensatory mechanisms have been so far demonstrated either in the immune system, or in the CNS [47]. It will be worth nevertheless verifying that the present conclusions remain true irrespective of the experimental conditions under which PrPc has been ablated. Another probable source of discrepancy is the heterogeneity of the T cell assay protocols. T cells were either assayed in a naive state, by polyclonal activation immediately after being collected ex vivo [30] or were in vitro challenged with antigen presenting cells and peptides a few days only after in vivo priming [31,32]. In contrast, T cells were presently probed three weeks after in vivo challenge with MOG_35-55 _peptide. At this time, a full-fledged response to MOG had developed in vivo, and EAE exacerbation in *Prnp*^-/- ^mice was clear-cut. If qualitative or quantitative differences in T cell responses were at the origin of disease worsening, they should have been evidenced at this point. The fact that the responses of PrPc-deficient T cells were modified at 7-10 dpi and appeared normal at 22 dpi suggests that the alterations in acquired immunity caused by PrPc-deficiency vanish with time. Nevertheless, the absence of PrPc on T cells may have a biological impact at the onset of some immune reactions as illustrated by the reduced resistance to sepsis of *Prnp*^-/- ^mice reported by Ingram and colleagues [30], and by our own data measuring in vivo T cell proliferation after only 6-day engraftment into PrPc-ablated mice [22]. Still, whether an early alteration of T-cell responsiveness may affect immune responses that develop in the longer term such as EAE requires further investigations. It should be emphasized that *Prnp*^-/- ^mice have been frequently used for the production of anti-scrapie hyperimmune sera and protective T cells [48], and that so far no obvious immunological anomaly was reported. More direct evidence for the implication of T cells in EAE exacerbation could be obtained by showing that mice adoptively transferred with encephalitogenic T cells that lack PrPc develop a more aggressive disease than mice transferred with PrP-sufficient T cells. Such experiments were not reported in any of the quoted studies. Our own attempts at transferring TCR transgenic 2D2 T cells generated in PrPc-positive and negative mice, gave results opposite to what might have been expected.

### Experimental BM chimeras support the implication of the CNS

The present data give support to a critical role of the radio-resistant cells of the CNS in EAE exacerbation. Chimeras with a PrPc-deficient immune system and a PrPc-sufficient CNS are less affected than reciprocal chimeras with a PrPc-positive immune system and a PrPc-deleted CNS. Disease begins earlier in the latter mice, reaches significantly higher clinical scores and progresses in a majority of mice toward ataxia and terminal stage sickness. Same differences were observed in irradiation controls restored with homologous BM. Disease in [*Prnp*^-/- ^>*Prnp*^-/-^] mice was definitively more aggressive than in [*Prnp*^+/+ ^>*Prnp*^+/+^] mice, but not more than in [*Prnp*^+/+ ^>*Prnp*^-/-^] chimeras, confirming that EAE exacerbation resulted primarily from PrPc absence in the CNS, and thus ruling out the possibility that the lack of PrPc in the lymphoid system might be a less prevalent, but still effective contributor to disease worsening.

### The impact of PrPc absence on glial cells can be partly dissociated from that on neurons

Currently, it is not possible to determine precisely whether exacerbation of EAE in a PrP-deficient context, originates from neuronal or glial dysfunction. Morphological analyses of cerebella from chimeras display simultaneously increased Purkinje neuronal degeneration visualized by a loss of PMCA2 staining reflecting early neuronal damage [45] and stronger glial activation assessed by GFAP and Iba-1 immuno-labeling. The reduced resistance of PrPc-deprived neurons to autoimmune assaults might precipitate gliosis via the release of apoptotic bodies or cytokines. Alternatively, the lack of PrPc in glial cells might enhance their phagocytic activity, as demonstrated for macrophages [28], or initiate an inflammatory cascade resulting in increased neurodegeneration. The cellular origin of PrPc deleterious effects being non-dissociable in radiation chimeras, it is impossible to evaluate precisely the contribution of each cell subset (astrocytes versus neurons). Studies that use transgenic mice with PrPc expression targeted to the different cell populations could shed light into this issue. Contrary to astrocytes, the impact of microglial PrPc can be distinguished from that of neuronal PrPc. Microglia is composed of blood borne macrophages that differentiate from BM progenitors and of resident microglial cells which have a potential of self-renewal within the brain [40,42,49]. Recent studies indicate that in chimeras conditioned by total body irradiation and restored with mechanically dispersed BM suspensions, as performed in this study, myeloid progenitors contribute to the pool of blood borne macrophages that replenish the resident microglia [50,51]. As microglia in [*Prnp*^+/+ ^>*Prnp*^-/-^] chimeras are mainly PrP-sufficient, we conclude that neither blood borne macrophages nor resident cells contribute to disease exacerbation. Thus, the hyperactived phenotype reflected by large accumulations of Iba-1 positive cells around inflamed vessels in [*Prnp*^+/+ ^>*Prnp*^-/-^] chimeras is probably secondary to neuronal damage.

### PrPc protects neurons under autoimmune assault

Finally, the most likely interpretation of EAE worsening in PrPc-ablated mice and in [*Prnp*^+/+ ^>*Prnp*^-/-^] chimeras is the absence of PrPc on neurons, a conclusion not totally unexpected in view of the abundant literature ascribing neuroprotective and neurotrophic functions to cellular prion protein [52,53]. Multiple mechanisms leading to neurodegeneration, axonal damage, demyelination, and oligodentrocyte death have been identified in EAE [33]. They include calpain-mediated caspase activation and apoptosis [54,55], oxidative stress [56], glutamate mediated excitotoxicity and calcium overload [57]. PrPc has the potential to interfere with all these pathways. PrPc protects neurons from Bax-mediated apoptosis [58], prevents caspase-induced mitochondrial apoptosis [59] and favors superoxide dismutase (SOD) activity [60,61], although the latter point has been disputed [62]. PrPc also down- regulates N-methyl-d-aspartate receptors on neurons and exerts a protective effect against subsequent glutamate-mediated toxicity [63]. All these actions of PrPc may protect against the damaging effects of cellular oxidation, especially in vulnerable neural cells such as neurons and oligodendrocytes. It is interesting to note that Shadoo, another member of the prion protein family [64], also expressed in the CNS and exerting stress-protective activity [65] does not compensate for the absence of PrPc in *Prnp*^-/- ^mice. A major future objective will be to further delineate the molecular pathways used by PrPc to protect the neurons from degeneration due to traumatic insults, autoimmune reactions or neurotoxic amyloid deposits.

## Conclusion

The biological function of PrPc and the evolutionary justification for its remarkable conservation in vertebrates are still not resolved. A plethora of candidate functions has been attributed to the prion protein, mainly in the nervous and the immune systems. Here we have examined the contribution of neural versus lymphoid PrPc in the pathogenesis of EAE, a disease model which develops at the confluence of the two systems. In contrast to previous reports inferring from T cell responses in vitro that disease worsening in PrPc-deficient mice had an immunological origin, we provide evidence, based on reciprocal BM chimera experiments, for the implication of CNS PrPc. These data highlight once more the critical role of PrPc in protecting the CNS from harming agents. They do not exclude the importance of lymphoid PrPc in immune responses, particularly at their onset, but they underline the possibility that evolution may have used the same gene for independent and equally important functions as for CD3zeta or semaphorins [66,67].

## Abbreviations

BM: Bone Marrow; B6: C57BL/6; CFA: Complete Freund's Adjuvant; CNS: Central Nervous System; DC: Dendritic Cell; dpi: days post inoculation; EAE: Experimental Autoimmune Encephalomyelitis; GFAP: Glial Fibrillary Acidic Protein; Iba-1: Ionized calcium-binding adapter molecule 1 (Iba1); MOG: Myelin Oligodendrocyte Glycoprotein; PBS: Phosphate Buffer Saline; PMCA2: Plasma Membrane Calcium ATPase2; PrPc: cellular Prion Protein; siRNA: small interfering Ribonucleic Acid; SOD: Superoxide Dismutase; TCR: T Cell Receptor; TSE: Transmissible Spongiform Encephalopathy.

## Competing interests

The authors declare that they have no competing interests.

## Authors' contributions

PG conceived experiments, carried out in vivo work and in vitro assays, participated to the analysis of data and helped to draft the manuscript. CB performed morphological studies, participated to the analysis of data and contributed to the manuscript. AN participated to the design and realization of the EAE experiments and histopathology, analyzed the data and drafted the manuscript. CC conceived the study, analyzed the data and drafted the manuscript. All authors read and approved the final manuscript.
